# Production Conditions Affect the *In Vitro* Anti-Tumoral Effects of a High Concentration Multi-Strain Probiotic Preparation

**DOI:** 10.1371/journal.pone.0163216

**Published:** 2016-09-22

**Authors:** Benedetta Cinque, Cristina La Torre, Francesca Lombardi, Paola Palumbo, Michel Van der Rest, Maria Grazia Cifone

**Affiliations:** 1 Department of Life, Health & Environmental Sciences, University of L’Aquila, Building Delta 6, Coppito, L’Aquila, Italy; 2 BioVisible BV, Groningen, The Netherlands; University of Palermo, ITALY

## Abstract

A careful selection of the probiotic agent, standardization of the dose and detailed characterization of the beneficial effects are essential when considering use of a probiotic for the dietary management of serious diseases. However, changes in the manufacturing processes, equipment or facilities can result in differences in the product itself due to the live nature of probiotics. The need to reconfirm safety and/or efficacy for any probiotic product made at a different factory is therefore mandatory. Recently, under the brand VSL#3®, a formulation produced by a manufacturer different from the previous one, has been commercialized in some European countries (the UK and Holland). VSL#3 is a high concentration multi-strain preparation which has been recognized by the main Gastroenterology Associations for the dietary management of pouchitis as well as ulcerative colitis. We have compared the “original” VSL#3 produced in USA with the “newfound” VSL#3 produced in Italy. According to our results, the “newfound” VSL#3 has 130–150% more “dead bacteria” compared to the “original” product, raising concerns for the well-known association between dead microbes with adverse effects. The abilities of bacterial lysates from the two formulations to influence *in vitro* viability and proliferation of different tumor cell lines also resulted different. The repair of previously scratched monolayers of various adherent tumor cell lines (i.e. HT1080, and Caco-2 cells) was inhibited more significantly by the “original” VSL#3 when compared to the “newfound” VSL#3. Tumor cell cycle profile, in particular cell cycle arrest and apoptotic death of the cancer cells, further confirms that the “original” VSL#3 has a better functional profile than the “newfound” VSL#3, at least in *in vitro*. Our data stress the importance of the production conditions for the “newfound” VSL#3 considering that this product is intended to be used for the dietary management of patients with very serious diseases, such as chronic inflammatory bowel diseases.

## Introduction

A probiotic is defined as a “*live microorganism which when administered in adequate amounts confers a health benefit on the host'* [1]. This definition underlines two important issues: firstly, the microbes in a probiotic formulation must be live, as it is the living bacteria that are beneficial to the host, and secondly, there is a dose-effect relationship with the health benefits depending on adequate doses. Thus, dose regimens and specifications for use must be clearly defined when probiotics are used in clinical practice. The following features are essential when selecting a probiotic product: i) the specific strain(s) must be characterized using appropriate phenotypic, and genotypic techniques [2] ii) the matrix in which the strains are delivered must be defined; iii) the number of live microorganisms provided in a given dose (expressed in CFU, Colony Forming Units) of the product must be specified (and should apply until the end of the product shelf-life), and, iv) the number of dead bacteria in the preparation must be within an acceptable range, for safety reasons.

To make any medical food claim, probiotics are required to demonstrate a beneficial effect through appropriate human clinical trials in the target patient group [3]. However, due to the nature of the product, i.e. live bacteria, any modifications in the formulation or in the manufacturing processes imply that the new product replacing the “original one” might be significantly different [4]. A change in manufacturing has the potential to influence efficacy and even safety of probiotics, independently of the genetic identity of the strains [5] and this aspect should be properly checked.

For the majority of the marketed probiotics, the available evidence deals mainly with their safety and ability to colonize the gut, rather than adequate clinical efficacy. On the contrary, for the VSL#3 formulation, a large body of clinical evidence has been reported for several groups of patients [6–10]. This formulation, containing a combination of 8 different strains of lactic acid bacteria and bifidobacteria, was specifically invented by Professor Claudio De Simone to achieve a unique biochemical and immunological profile, hereafter defined as “original” formulation. This “original” formulation has been endorsed for many years by numerous international Gastroenterological Societies, including the American Gastroenterology Association, and the European Crohn's and Colitis Organization, for the management of chronic pouchitis, the prevention of pouchitis after ileal-pouch anastomosis, as well as for maintenance treatment of ulcerative colitis [11]. Moreover, in 2015 the participants to a consensus session on the occasion of the 4^th^ Triennial Yale/Harvard Workshop on Probiotic Recommendations recognized for the first time level B and C evidence for this probiotic preparation in the management of non-alcoholic fatty liver disease (NAFLD) in adults and children, and in alcoholic liver diseases [12]. Manufacturing standards and quality consistency of the “original” formulation are therefore essential, considering the field of usage.

Recently, under the brand VSL#3®, a formulation produced by a manufacturer different from the previous one, has been commercialized in the UK and Holland, hereafter defined as “newfound” formulation. Our hypothesis was that even though the two formulations are claimed to be the same in terms of species identity and CFU counting, the biological effects of “original” and “newfound” VSL#3 when compared *in vitro* on tumor cell lines’ viability, proliferation, cell cycle profile and apoptotic death levels would be significantly different.

## Materials and Methods

### “Original” and “newfound” VSL#3

VSL#3 is a composite probiotic mixture containing multiple strains of three viable lyophilized bacteria species: three strains of *bifidobacteria* (*B*. *longum* DSM 24736, *B*. *infantis* DSM 24737 and *B*. *breve* DSM 24732), four strains of lactobacilli (*L*. *acidophilus* DSM 24735, *L*. *paracasei* DSM 24733, *L*. *bulgaricus* DSM 24734 and *L*. *plantarum* DSM 24730), and one strain of *Streptococcus salivarius* subspecies *thermophilus* DSM 24731. Some boxes of VSL#3® distributed by Ferring Pharmaceuticals were purchased in the UK (lot number 507132, expiry date 07/2017; “newfound” VSL#3) and also in Holland (lot number 512058, expiry date 12/2017) for testing and comparison with the product distributed by Ferring in Italy (lot TM091; expiry date 10/2017; “original VSL#3”). The Italian product we analyzed was produced in the USA, while the NL and UK products were manufactured in Italy.

**Fig 1:**
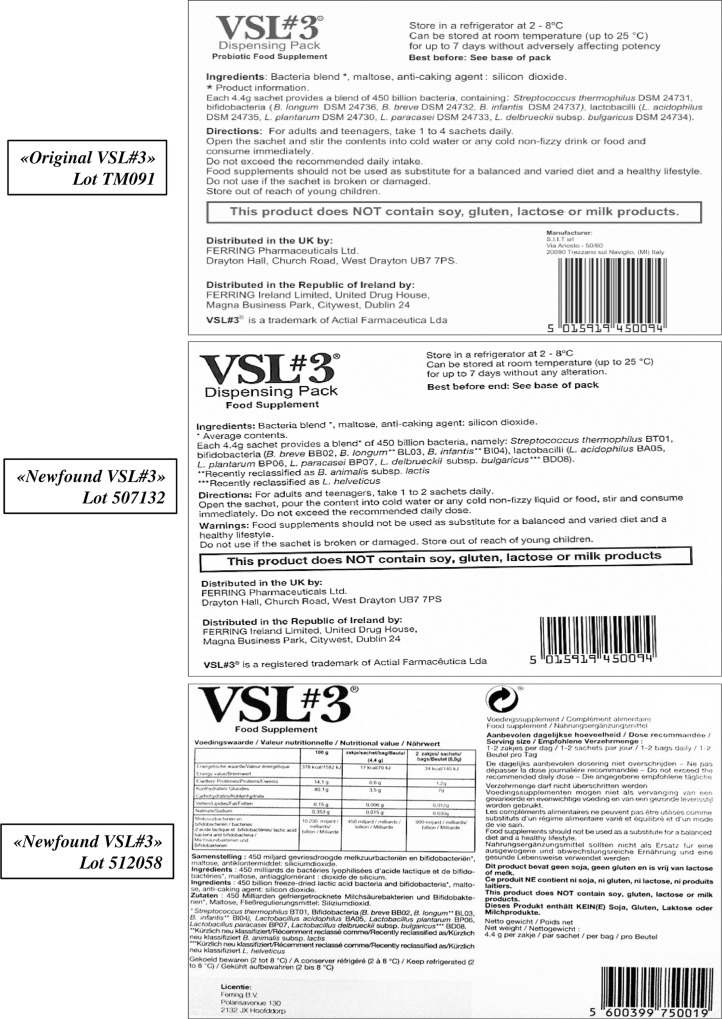
“Original” and “newfound” VSL#3 formulations.

No genetic comparative strain analysis was performed between the two formulations available under the same brand VSL#3®, trusting what declared by Ferring Pharmaceuticals that only the strain designation has been updated and modernized, without any change in the product. Consequently the list of the strains present in the “original” VSL#3 as it appears on the box, is: *Streptococcus thermophilus* DSM 24731, bifidobacteria (*B*. *longum* DSM 24736, *B*. *breve* DSM 24732, *B*. *infantis* DSM 24737), lactobacilli (*L*. *acidophilus* DSM 24735, *L*. *plantarum* DSM 24730, *L*. *paracasei* DSM 24733, *L*. *debrueckii* subsp. *bulgaricus* DSM 24734), while the composition of the “newfound” VSL#3 formulation is reported on the package as: *Streptococcus thermophilus* BT01, bifidobacteria (*B*. *breve* BB02, *B*. *longum* BL03, *B*. *infantis* BI04), lactobacilli (*L*. *acidophilus* BA05, *L*. *plantarum* BP06, *L*. *paracasei* BP07, *L*. *debrueckii* subsp. *bulgaricus* BD08) (Fig 1).

### Counting of live/dead bacteria from “original” and “newfound” VSL# formulations

The live/dead status of bacteria in the VSL#3 product was assessed using a mixture of SYTO^®^13 green fluorescent nucleic acid stain (Molecular Probes, Inc., Eugene, OR) and the red fluorescent nucleic acid stain, Propidium Iodide (PI) (Life Technologies, Europe). These stains differ both in their spectral characteristics and in their ability to penetrate healthy bacterial cells. When used alone, the SYTO^®^13 stain labels bacteria with both intact and damaged membranes. In contrast, PI penetrates only bacteria with damaged membranes, competing with the SYTO^®^13 stain for nucleic acid binding sites when both dyes are present. When mixed in correct proportions, SYTO^®^13 stain and PI produce green fluorescent staining of bacteria with intact cell membranes and red fluorescent staining of bacteria with damaged membranes. The background remains virtually non-fluorescent. The standard LIVE/DEAD viability assay (Life Technologies, Europe) uses SYTO^®^9 instead of SYTO^®^13, in this case SYTO^®^13 is used as an alternative since SYTO^®^13 proved to have less background in the analysis. 100 mg VSL#3 product was dissolved in 1 ml H_2_O. 2 μl of the VSL#3 mix was added to 100 μl of staining solution containing 10 μM PI and 8 μM SYTO^®^13 and stored in the dark at room temperature for 15 minutes. After the incubation the solution was diluted in 1 ml H_2_O and applied on a glass slide. BacLight™ mounting oil (Life Technologies, Europe) was applied, and cells were inspected visually using an Olympus BX60 epifluorescence microscope using a FITC or Cy3-specific filter. Live cells stain green and dead cells stain red. Different amounts of cells were applied on the glass slide and 10 microscope views were analyzed.

### Preparation of bacterial lysates for cell cultures

Stocks of 1 g of each VSL#3 lot were suspended in 10 ml of PBS. After three freeze-thaw cycles, samples were sonicated (30 min, alternating 10 s of sonication and 10 s of pause) with a Vibracell sonicator (Sonic and Materials, Danbury, CT). Bacterial cells disruption was verified by measuring the absorbance of sample at 590 nm before and after every sonication step. The sonication steps were repeated until the optical density reached a constant value. Protein concentration was measured using Bio-Rad Dc Protein Assay (Bio-Rad, Hercules, CA USA). The total protein level was comparable in all bacterial lysates’ samples (range 5–6 mg protein/ml). For the *in vitro* experiments, cell lines were treated for different times as below specified, depending on the type of experiment, with the bacterial lysates’ suspensions at 10, 50 and 100 μl/ml (final concentration), corresponding, respectively to 1, 5 and 10 mg (lyophilised bacteria weight)/ml. According to the manufacturer’s informations, the amounts of bacterial lysates added to the cell cultures corresponded, respectively, to 10^8^, 5x10^8^ and 10^9^ CFU/ml culture medium.

### Cell lines and culture conditions

Acute human T leukemia-derived Jurkat cell line and human fibrosarcoma HT1080 cell line were acquired from ATCC (American Type Culture Collection, Georgetown, DC, USA); human colon adenocarcinoma-derived Caco-2 cell line were purchased from Sigma-Aldrich (St. Louis, MO, USA). Jurkat cells were routinely grown in plastic culture flasks containing RPMI 1640 medium supplemented with 2% FCS, 2 mM L-glutamine, penicillin (100 U/ml), and streptomycin (100 μg/ml). Subculturing was performed every 2–3 days and the culture medium was totally replaced by centrifugation for 10 min at 400*xg*. Jurkat cells were plated 5x10^5^ cells for each condition. HT1080 cells were cultured in DMEM supplemented with 10% (v/v) of FCS and with 2 mM L-glutamine, 100 U/ml penicillin and 100 μg/ml streptomycin in a monolayer culture. HT1080 cells were seeded into a sterile tissue culture 12-well plate (Becton Dickinson, Franklin Lakes, New Jersey, USA) at 18,000 cells/cm^2^ for each experimental condition. Caco-2 cells were maintained in culture in DMEM supplemented with 10% FCS, 1% non-essential amino acid, 1 mM sodium pyruvate and 2 mM L-glutamine, 100 U/ml penicillin and 100 μg/ml streptomycin and seeded at 18,000 cells/cm^2^ for each experimental points. Under these culture conditions, this cell line remained proliferative and undifferentiated. For adherent cells, after reaching 80% confluence adherent cell cultures were expanded using a trypsin solution from bovine pancreas (Sigma-Aldrich). The well plates were incubated in sterile conditions at 37°C in a 5% CO_2_ humidified atmosphere. The complete medium was totally replaced every three days. All culture reagents were acquired from Euro Clone (West York, UK).

### Cell viability and proliferation assay

Cultured cells were collected at 12, 24 and 48 hours and live cells were counted after trypan blue dye (Euro Clone, West York, UK) exclusion. Cells were incubated with bacterial lysates at 1, 5 or 10 mg/ml, as above described, for 12–48 h, after which were washed with PBS, centrifuged for 10 min at 400*xg* and the pellets incubated with a 0.04% trypan blue solution for 5 min. Afterwards, cells were transferred to a Bürker counting chamber and counted by microscopy (Eclipse 50i, Nikon Corporation, Japan). Dead cells looked totally blue stained and living cells appeared normally bright and colourless. The cell number counts as well as the percentage of live and dead cells were thus registered. Non-treated cells were also analyzed and served as negative controls.

### *In vitro* monolayer wound healing assay and image processing methods

As previously described [13], for the *in vitro* monolayer wound healing assay, cell cultures were allowed to proliferate until ~90% confluence was reached, then DMEM was removed from the well and cell monolayers were scratched using a 200 μl pipet tip to create a uniform cell-free wound area with reproducible width of wounding (0.7 mm width). Debris was removed from the culture by gently washing with sterile PBS. Cell cultures were incubated with fresh medium at 37°C in a 5% CO_2_ humidified atmosphere in the presence or absence of bacterial lysates at 1, 5 or 10 mg/ml, as above described. Non-treated cells were also analyzed and served as negative controls.

Cell migration was monitored by microscopy using an inverted light microscope (Eclipse TS 100, Nikon) and photographed from time 0 (T0), after 15 hours (T15h), and 22–24 hours. The last time point to observe the total scratched monolayer closure after the injury in the untreated cells was in the range 24–28 hours. The images acquired for each sample were further analyzed quantitatively as illustrated below. The amount of wound closure by cell migration consisting in calculation of percentage of closure of the wound was performed using TScratch, a software tool developed to automatically analyze wound healing assays. It is available as a stand-alone application using Matlab [14].

### Cell cycle and apoptosis analysis by flow cytometer

For cell cycle and apoptosis analysis, Jurkat, HT1080 and Caco-2 cell lines were treated for 24 or 48 h with 10 mg/ml bacterial lysate. After treatment, the cells were collected, washed with PBS, collected by centrifugation at 400*xg* for 10 min at 4°C, counted and fixed adding of cooled ethanol solution at 70% in PBS with gentle mixing at 4°C for 30 min. Fixed cells (10^6^ cells/ml) were transferred to plastic BD tubes (Becton Dickinson, San Jose, CA), washed twice with ice-cold PBS and stained with solution containing 50 μg/ml Propidium Iodide, 0.1% Nonidet-P40 and RNase A (6μg/10^6^ cell) for 30 min in the dark at 4°C. Cell cycle phase-distribution was analyzed by a flow cytometry system. Data from 10,000 events per sample were collected and analyzed using FACS Calibur (Becton Dickinson) instrument equipped with cell cycle analysis software (Modfit LT for Mac V3.0). Apoptotic cells were also determined by their hypochromic subdiploid staining profiles and analyzed using Cell Quest software (BD Biosciences). Non-treated cells were also analyzed and served as negative controls.

In addition, the detection of nucleic acid-containing microparticles generated in vitro from cell lines through SYTO^®^13 (Molecular Probes, Inc., Eugene, OR) staining was also used to reveal apoptotic cell death level, as previously described [15]. Treated cells were incubated for 48 h with bacterial lysates, as above described. The media were collected and used for analysis of microparticles by flow cytometry. The cell suspensions were centrifuged at 300x g for 5 min at room temperature to pellet out the cells and obtain the cell-free supernatants containing microparticles. A fifty μl aliquot of the microparticle suspensions was diluted 1:8 in PBS or in 200 nM solution of SYTO^®^13 and directly used for flow cytometry. Analysis was carried out using Cell Quest software (BD Biosciences).

### Oligonucleosome assay

DNA fragmentation was measured by quantitation of cytosolic oligonucleosome-bound DNA, using an enzyme-linked immunosorbent assay (ELISA) kit (Cell Death Detection Plus ELISA; Roche), according to the manufacturer's instructions. This assay determines apoptosis by measuring mono- and oligonucleosomes in the lysates of apoptotic cells. Briefly, after treatment, the cells were collected and washed with PBS, then the cytosolic fraction was extracted by centrifugation at 200*xg*. Cytosolic fractions were placed in duplicate into wells of streptavidin-coated microplate, to which was added the immunoreagent containing a mixture of anti–histone-biotin and anti–DNA-POD. The plate was incubated for 2 hours at room temperature on a MP shaker at 300 rpm. During the incubation period, the anti-histone antibody binds to the histone component of the nucleosomes and simultaneously captures the immunocomplex to the streptavidin-coated microplate via its biotinylation. At the same time, the anti–DNA-POD antibody reacts with the DNA component of the nucleosomes. The unbound antibodies were washed with incubation buffer. The amount of nucleosomes retained by the POD in the immunocomplex, corresponding to the extent of apoptosis was determined photometrically with ABTS (2,2’-azinobis-3-ethyl-benzothiazoline-6-sulfonic acid) as substrate using microplate reader at a wavelength 405 nm and reference wavelength of 490 nm. Obtained data were expressed as Arbitrary Unit/10^6^ cells.

### Statistical analysis

Data (mean ± SD) were analyzed using Prism 5.0 GraphPad Software, San Diego, Ca. For comparison between two means, Student’s unpaired t test was used. For comparison of groups, a one or two-way ANOVA test followed by Bonferroni post hoc test were used. *P<*0.05 was considered statistically significant.

## Results

### Lot identification and source of bacteria

The boxes containing the product were handled according to the manufacturer’s instructions. The box purchased in Italy (lot TM091, expiry date 10/2017) contained the probiotic mix produced in the USA, a formulation already utilized by our group for previous publications in 2001, 2008 and 2011 and other unpublished tests [16–18] (“original” VSL#3). The box purchased from the UK (lot 507132, expire data 07/2017) and the box purchased from Holland (lot 512058, expiry date 12/2017) contained the probiotic mix produced in Italy (“newfound” VSL#3). These products are distributed in all three countries by the same group Ferring. Since, as evidenced further, the UK and Holland lots manufactured in Italy had both very similar and comparable characteristics, markedly different from the Italian product manufactured in the US, some of the comparative tests were performed only with the UK product. No other lots were examined during the preparation of this manuscript, due to the fact that this product has a 2 year shelf life, and the authors do not know the quantities produced for each lot, nor the levels of sales, and therefore are not in a position to envisage the time when new lots would be commercially available and therefore available for testing.

### Live versus dead bacteria in “original” vs “newfound” VSL#3 product

The proportion of live to dead cells in a bacterial formulation is a key element and has a material effect on the formulation’s efficacy. Both the “original” and the “newfound” VSL#3 formulations are labelled on their packaging as “Live freeze-dried bacteria, 450 billion bacteria per sachet.” When the number of bacterial cells in the preparation is expressed as CFU (Colony Forming Units) the number of dead bacteria present in the product is not disclosed.

**Fig 2:**
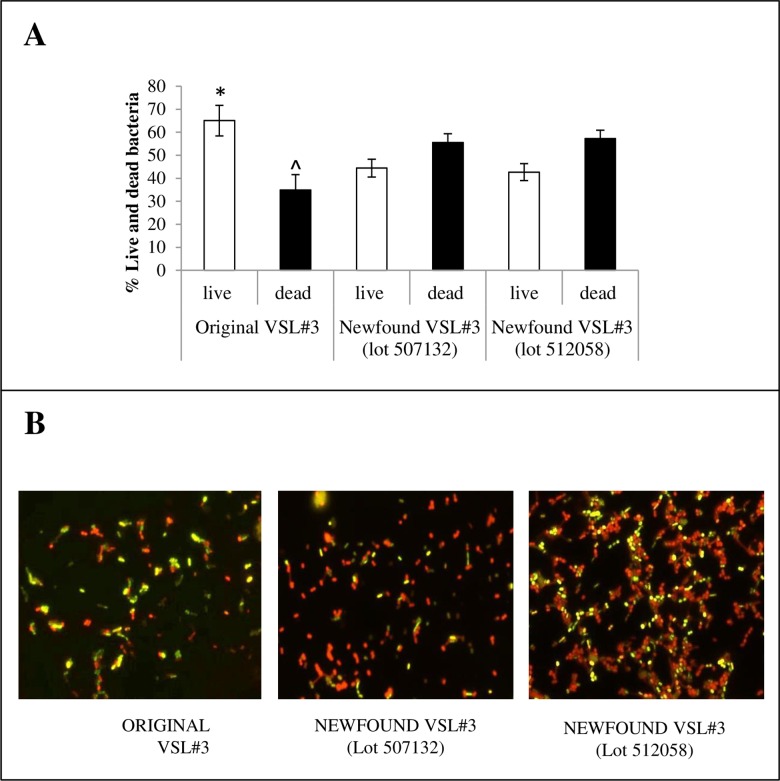
Live vs dead bacteria in “original” and “newfound” VSL#3 product. Panel A: The percentages of live and dead cells in each VSL#3 formulations presented as mean ± SD of n. 10 determinations are shown. (Live cells *P<0.001 vs both lots of newfound formulations; Dead cells ^*P*<0.001 vs both lots of newfound formulations). Panel B: Representative microscopy images of bacteria stained with SYTO^®^13 and Propidium Iodide are shown. Stained bacterial suspensions were analyzed using a fluorescent microscope. Red colour is due to SYTO^®^13 green fluorescence that was quenched by that of PI (dead bacterial cells), while bright green-yellow colour was due to SYTO^®^13 staining when PI did not permeate across the cell membrane (live bacterial cells). Magnification 100x.

Our analysis checked the percentage of “live” bacteria versus “dead” bacteria in a sachet of each formulation. Live cells in probiotic products will inevitably lose viability, and the actual products will contain varying amounts of dead cells. In practice, it is technically not possible to administer only live bacteria to a subject. Since the “dead” bacteria affect the responses of the individual, controlling the quality of the product simply by counting the “live” bacteria is seriously questionable. The percentage of “live” and “dead” bacteria in a sachet is an important parameter since: (a) the capability of the product to colonize the gut properly is related to the number of “live” bacteria per sachet; (b) the quantity of “dead” bacteria is a good predictor of the quality of the product and its stability, and (c) “dead” bacteria are not an inert material. Both lots of “newfound” product contained a considerably lower percentage of live cells (*P<0*,*0001*) and a markedly higher percentage of dead cells (*P<0*.*0001*) when compared to the “original” VSL#3 formulation (Fig 2A). Accordingly, the ratio live/dead bacterial cells resulted significantly higher in “original” VSL#3 vs both lots of the “newfound” formulation (*P*<0.0001). These differences can be clearly visualized from the microscopy images (Fig 2B).

Consequently, in addition to the declared 450 billion live bacteria (CFU), each sachet of “original” VSL#3 will contain 242 billion dead bacteria (450 billion corresponds to the 65.08% live bacteria, thus 34.92% gives 242 billion of dead bacteria as reported in Fig 2A). In the same way of calculation, it turns out that both lots of the “newfound” product contain 560–600 billion of dead bacteria in addition to the 450 billion live bacteria. Each single sachet of the “newfound” product has 130–150% more dead bacteria than the “original” product and this may significantly affect the response and health status of the person ingesting it, especially children [19].

### Effects of “original” and “newfound” VSL#3 bacterial lysates on tumor cells’ viability and proliferation *in vitro*

**Fig 3:**
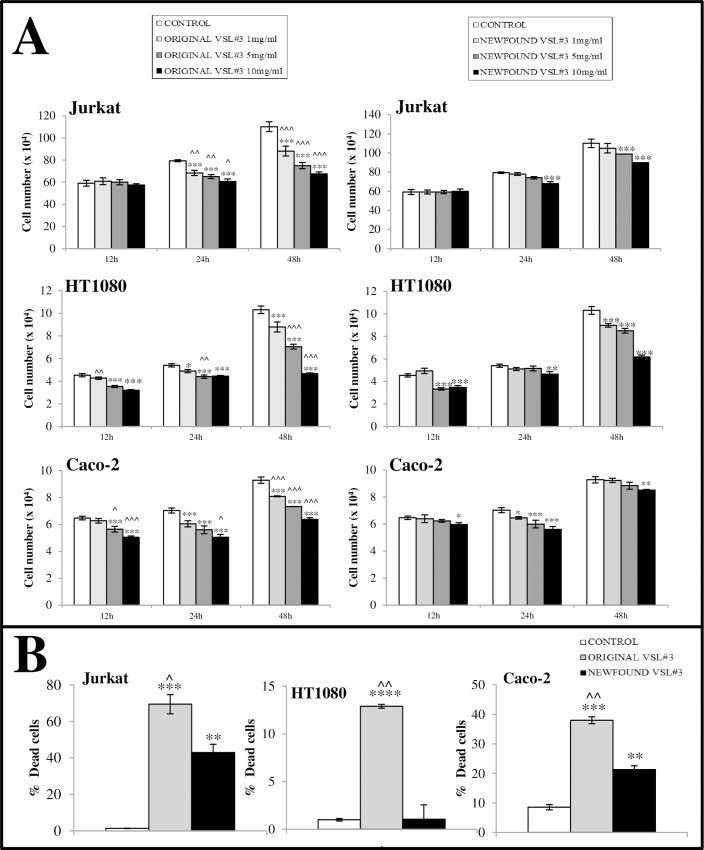
**A) Comparison of the effects of “original” and “newfound” VSL#3 formulations on tumor cell growth.** The cells were cultured for 12, 24 or 48 h in the presence or absence of probiotic lysates at 1, 5 and 10 mg/ml. The results from one representative from n. 3 independent experiments are presented as the mean values of duplicates ± SD. *P<0.05; **P<0.01; ***P<0.001 vs untreated control; ^*P*<0.05; ^^*P*<0.01; ^^^P<0.001 vs “newfound VSL#3” lysate treatment. All data shown are representative of 3 independent experiments. **B) Comparison between the effects of bacterial lysates from each VSL#3 formulation on cell death % level on tumor cell lines.** Dead cell number was assessed by Trypan blue dye inclusion after 48 h treatment *in vitro*. Percentages of dead cell number are presented as mean of duplicates. *P<0.05; **P<0.01; ***P<0.005; ****P<0.001 vs untreated control; ^*P*<0.05; ^^*P*<0.01 vs “newfound VSL#3” lysate treatment. All data shown are representative of 3 independent experiments.

The effect of the treatment at 12, 24 or 48 h with bacterial lysates from “original” and “newfound” VSL#3 on cell growth, viability and death was assayed by Trypan blue dye exclusion test. Jurkat, HT1080, and Caco-2 tumor cell lines were differently affected by *in vitro* treatment, in a time and dose-dependent way being the “original” VSL#3 treatment in general more efficient in inducing anti-proliferative effect when compared to “newfound” VSL#3/lot 507132 (Fig 3A). The results obtained with bacterial lysates from lot 512058 were similar to those obtained with lot 507132 of “newfound” VSL#3.

Accordingly with previous reports which showed the ability of some probiotics to exert antitumor effects through different potential mechanisms [20–23], both “original” and “newfound” VSL#3 lysate samples at 10 mg/ml were able to significantly reduce after 48 h the number of viable cells in the analyzed tumor cell lines, thus confirming their ability to induce both inhibition of proliferation and cell death. However, in these experimental conditions, the inhibition of viable cell number increase observed after treatment with “newfound” VSL#3 was significantly lower when compared to that observed with the “original” product for all used cell lines. Trypan blue dye inclusion assay showed a different sensitivity of tumor cells to bacterial lysate treatments in terms of dead cell percentage being Jurkat cells more negatively affected by treatment when compared to HT1080 or Caco-2 cells. The bacterial lysates from “original” VSL#3 product in every condition was more efficiently able to induce tumor cell damage in comparison to the “newfound” VSL#3 product which, in turn, resulted unable to exert any cell toxicity on HT1080 cells and able to induce a cell death levels in Jurkat or Caco-2 cells significantly lower respect to those observed after treatment with “original” VSL#3. Percentages of Jurkat, HT1080 and Caco-2 dead cells after 48 h treatment with 10 mg/ml bacterial lysates from a representative experiment are shown in Fig 3B. The results obtained with bacterial lysates from lot 512058 were similar to those obtained with lot 507132 of newfound product.

### Comparison between “original” and “newfound” VSL#3 formulations’ effects on scratched monolayer wound healing of tumor cell lines

**Fig 4:**
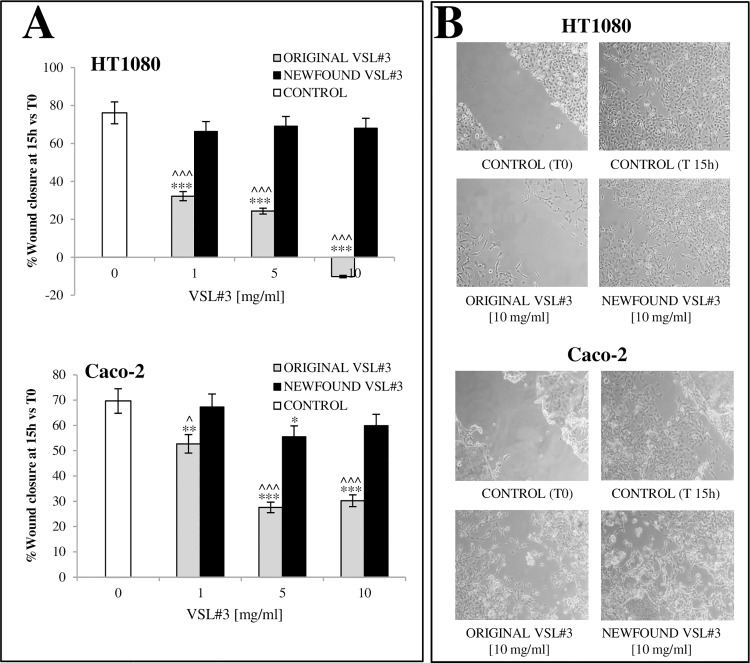
**A) Comparison between the effects of bacterial lysates on proliferation/migration ability of tumor cells assayed through *in vitro* “wound healing model”.** The effect of bacterial lysates at 1, 5, and 10 mg/ml on the resurfacing of scratched monolayers was analyzed and compared to the relative control cells. The microscopy images acquired for each sample were analyzed with TScratch software to automatically quantify the percentage of wound closure. Data are presented as the mean of duplicate values ± SD) and are relative to % wound closure at 15 h from monolayer scratching. The results shown are representative of 3 independent experiments. *P<0.05, **P<0.01, and ***P<0.001 vs untreated control; ^*P*<0.05 and ^^^*P*<0.001 vs “newfound VSL#3” lysate treatment. **B) Representative microscopy images of monolayers after treatment with “original” and “newfound VSL#3” formulations on adherent tumor cells at 15 h (T15) after scratching of monolayer.** An image of scratched untreated monolayer for each used cell line at T0 is also shown. Images from one representative out of 3 independent experiments are shown.

In order to further compare the ability of “original” and “newfound” VSL#3 to influence cell proliferation and migration, an *in vitro* artificial wound model was used on adherent tumor (HT1080, Caco-2) cell lines. The effect of bacterial lysates at 1, 5, and 10 mg/ml on the rate of closure of scratched monolayers was analyzed and compared to the relative untreated cells, as described in Methods’ section. The wound closure (percentage of closure), in either untreated or bacterial lysate-treated cells, was evaluated by observing in microscopy the re-populating area between the wound margins at different time intervals after the lesion. In order to quantitatively analyze the effects of bacterial lysates on wounded area resurfacing, the images obtained by inverted light microscope proliferation were acquired and converted to % closure by using a mathematical system able to automatically calculate the portion of area occupied by the cells. In Fig 4A are shown the results from a representative from 3 independent experiments. Data are presented as the mean of duplicate values ± SD and are relative to % wound closure at 15 h from monolayer scratching. The treatment with the “original” VSL#3 lysate led to a relevant and statistically significant inhibition of monolayer repair process which appeared strictly dose-dependent in HT1080 and only partly dose-dependent on Caco-2 tumor cell lines. On the other hand, “newfound” VSL#3 bacterial lysate, which didn’t significantly influence HT1080 monolayer resurfacing, induced just a weak and dose-independent inhibition on wound closure which was strongly less relevant when compared to that induced by “original” VSL#3 sample. Representative images from microscopic observations of scratched monolayers untreated (control) or treated with bacterial lysates at 15 h from injury are shown in Fig 4B. An image from untreated cells at T0 is also shown.

### Comparison between “original” and “newfound” VSL#3 formulations’ effects on cell cycle and apoptosis level of tumor cell lines

To further comparatively investigate the *in vitro* biological effects of “original” and “newfound” VSL#3 bacterial lysates, their ability to influence cell cycle distribution and apoptosis level on tumor cell lines was also assessed. In Table 1 are reported the results from a representative experiment out of 3 independent experiments in duplicate aimed to analyze the cell cycle distribution after 24 h treatment with “original” or “newfound VSL#3” bacterial lysates (10 mg/ml).

**Table 1:** Effect of 24 h treatment with “original” or “newfound” VSL#3 bacterial lysates (10 mg/ml) on cell cycle distribution (%).

Cell line	Treatment	G0/G1	S	G2/M
**Jurkat**	**Not treated (Control)**	**53.34±1.76**	**32.55±1.43**	**14.11±0.61**
	**“ORIGINAL VSL#3”**	**59.03±1.95**^**a**^	**35.15±1.75**	**5.82±0.29**^**b**^
	**“NEWFOUND VSL#3”**	**53.54±1.28**	**34.19±1.71**	**12.35±0.52**
**HT1080**	**Not treated (Control)**	**49.96±1.67**	**31.89±0.94**	**18.1±2.54**
	**“ORIGINAL VSL#3”**	**35.27±0.88**^**c**^	**59.25±0.92**^**d**^	**5.38±0.06**^**d**^
	**“NEWFOUND VSL#3”**	**36.79±0.82**^**e**^	**48.19±0.98**^**e**^	**15.01±0.15**
**Caco-2**	**Not treated (Control)**	**56.05±2.08**	**31.67±2.74**	**12.29±0.66**
	**“ORIGINAL VSL#3”**	**54.34±0.47**	**39.95±1.20**^f^	**5.72±0.74**^**g**^
	**“NEWFOUND VSL#3”**	**57.28±1.81**	**32.21±0.23**	**10.57±1.51**

^a^ *P<0*.*05* vs control; *P<0*.*01* vs NEWFOUND

^b^ *P<0*.*01* vs control; *P<0*.*05* vs NEWFOUND

^c^ *P<0*.*001* vs control; *P<0*.*05* vs NEWFOUND

^d^*P<0*.*001* vs control; *P<0*.*001* vs NEWFOUND

^e^ *P<0*.*001* vs control

^f^ *P<0*.*01* vs control; *P<0*.*01* vs NEWFOUND

^g^ *P<0*.*01* vs control; *P<0*.*05* vs NEWFOUND

Tumor cells appeared to be differently affected by treatment with bacterial lysate samples. Cytofluorimetric analysis indicated that a G0/G1 cell cycle arrest and a parallel G2/M percentage reduction were induced in Jurkat cells by addition of “original” VSL#3 lysate. This effect was statistically significant either when compared to untreated cells or to “newfound” VSL#3 sample. On the other hand, the “newfound” VSL#3 formulation didn’t significantly influence the Jurkat cell cycle distribution.

The treatment with “original” VSL#3 bacterial lysate of both HT1080 and Caco-2 tumor cells led to a significant increase of the S phase cells with a parallel reduction of both G0/G1 and G2/M phase %. The results were statistically significant when compared either to untreated cells and “newfound” VSL#3-treated cells. The effect of “newfound” VSL#3 formulation even if able to influence with a similar trend the cell cycle distribution, was definitely much weaker as compared to that obtained with the “original” product. Indeed, a statistically significant difference was observed between “original” and “newfound” VSL#3 effects on cell cycle distribution either on HT1080 or Caco-2 cells.

**Fig 5:**
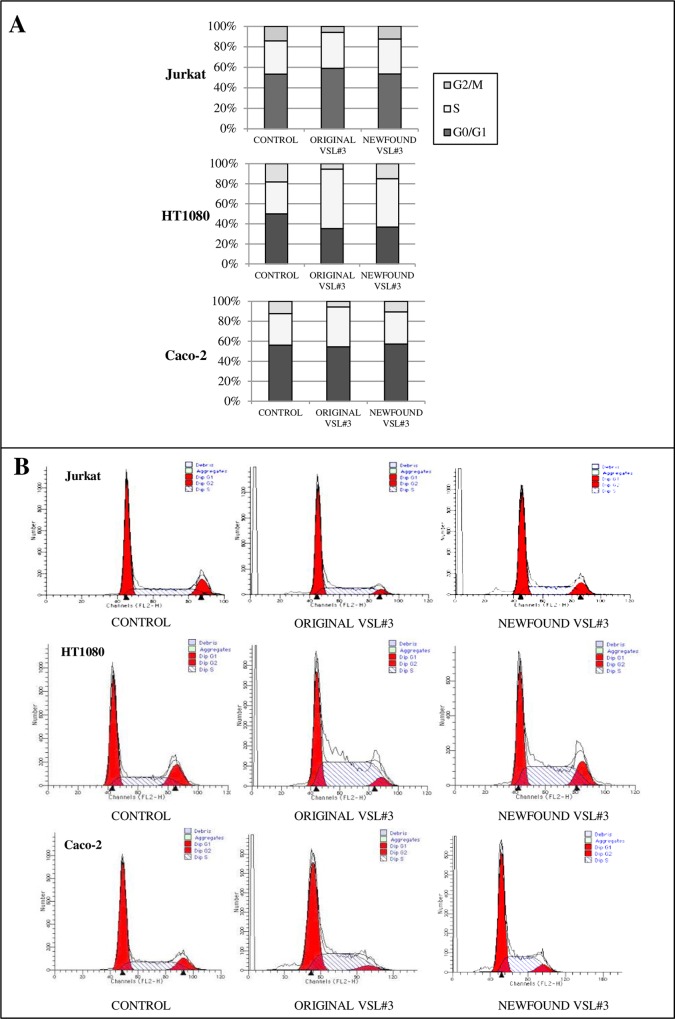
Effect of bacterial lysates from “original” or “newfound VSL#3” products on tumor cell cycle distribution. A) Histograms showing percent distribution of G0/G1, S, and G2/M cycle phase of tumor cells after 24 h treatment with probiotic lysates at 10 mg/ml. B) Representative cell cycles profiles of tumor cell lines after the same culture conditions above described.

The results listed in Table 1 are also shown as histograms in Fig 5A. Cytofluorymetric profiles from a representative experiment aiming at evaluating the effects of both “original” and “newfound” VSL#3 bacterial lysates on all used tumor cell lines are shown in Fig 5B.

**Fig 6:**
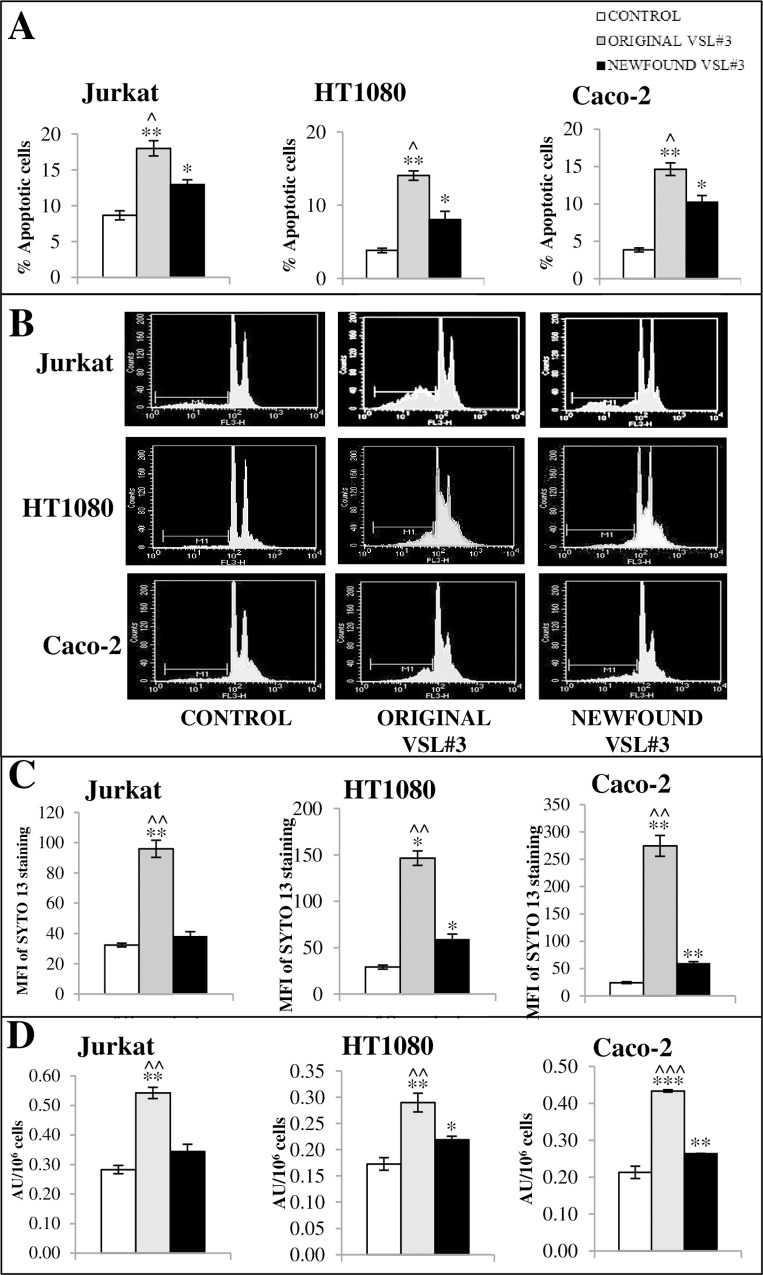
Effect of bacterial lysates from “original” or “newfound VSL#3” products on tumor cell apoptosis level. A) Data from cytofluorimetric analyses of apoptotic cells after treatment with bacterial lysates at 10 mg/ml for 48 hr expressed as mean % apoptotic cells ± SD. The data are relative to one representative out of n. 3 experiments in duplicate. *P<0.05, **P<0.01 vs untreated control; ^*P*<0.05 vs “newfound VSL#3” lysate treatment. B) The cytofluorimetric profiles from one representative from n. 3 experiments are shown. C) SYTO 13 staining of nucleic acid-containing microparticles generated in vitro from cell lines. Treated cells were incubated for 48 h with bacterial lysates, the media were collected and used for analysis of microparticles by flow cytometry. MFI, Mean Fluorescence Intensity. D) DNA fragmentation assay as measured by quantitation of cytosolic oligonucleosome-bound DNA, using an enzyme-linked immunosorbent assay (ELISA). Data from oligonucleosome assay after treatment with bacterial lysates at 10 mg/ml for 48 hr are expressed as Arbitrary Unit/10^6^ cells (mean of duplicate values ± SD). The data are relative to one representative out of n. 3 experiments in duplicate. *P<0.05, **P<0.01, and ****P<0.001 vs untreated control; ^^*P*<0.01, and ^^^*P*<0.001 vs “original VSL#3” lysate treatment.

In order to examine whether “original” and “newfound” VSL#3 bacterial lysates can promote apoptotic death in our tumor cell systems, cell death was monitored by cytofluorimetry, as described in the Methods section. In our experimental conditions, Jurkat, HT1080, and Caco-2 tumor cells appeared to be all influenced, even if at different levels, by treatment for 48 h with “original” and “newfound” VSL#3 bacterial lysates at 10 mg/ml. “Original” VSL#3 product resulted more efficient in inducing a statistically significant increase of apoptosis level in all analyzed tumor cell lines when compared either to untreated cells or “newfound” VSL#3 bacterial lysate. Fig 6A shows the results of cytofluorimetric analyses of all used cell lines, expressed as mean % apoptotic cells ± SD from one representative from n. 3 experiments in duplicate. Fig 6B shows the apoptosis cytofluorimetric profiles from one representative of n. 3 experiments. These results were confirmed by SYTO 13 staining of nucleic acid-containing microparticles in the cell-free supernatants, as shown in Fig 6C. The ability of bacterial lysates to induce apoptotic death of tumor cells was also verified through DNA fragmentation assay by oligonucleosomal ELISA. The results confirmed the higher effectiveness of “original” VSL#3 formulation as compared to “newfound” VSL#3 in inducing oligonucleosome formation (Fig 6D).

## Discussion

VSL#3, a multi-species probiotic mixture, is widely used clinically, mainly to treat mild to moderately active inflammatory bowel disease [24]. It also appears to be beneficial in patients with advanced liver disease by decreasing plasma levels of proinflammatory cytokines (e.g. TNFα) [25], improving quality of life [26] and reducing hospitalizations for hepatic encephalopathy [27]. VSL#3 assumption was also recently linked to good effects on brain function [28, 29].

While the “original” VSL#3 formulation is not regulated as a drug, the guidance of the FDA with respect to biologic products is nevertheless relevant. As the FDA notes repeatedly in numerous guidance documents: “In contrast to chemically synthesized small molecular weight drugs, which have a well-defined structure and can be thoroughly characterized, biological products are generally derived from living material-human, animal, or microorganism- are complex in structure, and thus are usually not fully characterized” [30]. The FDA further notes: “Because, in many cases, there is limited ability to identify the identity of the clinically active component(s) of a complex biological product, such products *are often defined by their manufacturing processes*. Changes in the manufacturing process, equipment or facilities could result in changes in the biological product itself and sometimes require additional clinical studies to demonstrate the product's safety, identity, purity and potency.” Sanders et al. [4] have analyzed thoroughly the possible effects of genetic and processing changes on efficacy and safety of probiotics, highlighting some key issues to determine the need to re-confirm efficacy and safety aspects of probiotics, in particular after manufacturing process changes, both from a scientific and regulatory standpoint. It is well accepted that growth conditions, growth substrates, cryoprotectants, food formulation and storage conditions may affect the properties of the probiotic, thereby generating changes in gene expression and possibly in metabolic output. Changes can have an impact on the numerical recovery of the probiotic and its biochemical and immunological profiles and may consequently influence the final health outcome. As an example, in one study *Bifidobacterium animalis* subsp. *lactis* INL1 [31] was grown on a particular culture deMan Rogosa Sharpe broth (MRS) at two different pH levels (6.5 and 5.0). When the final cultures were subjected to gastric resistance testing, the viability loss for the cells grown at pH 6.5 was a dramatic *4 logs higher* than for the cells grown at pH 5.0. Additionally, further analysis by electron microscope found that the culture grown at pH 6.5 showed production of extracellular compounds (exopolysaccharide type) not found in the same strain grown at pH 5.0. Authors found that these extracellular compounds could exert an *in vivo* response not found with the strains grown at lower pH. pH sensitivity of a given probiotic strain appears to be not only influenced by the pH level in culture production but also by the type of cryoprotectant used for freeze-drying of strains. Saarela *et al* (2009) [32] showed that resistance to gastric acidity and bile salts by a strain of *Lactobacillus rhamnosus* was variable depending not only on the fermentation pH level but also on the use of either polydextrose vs sucrose as a cryoprotectant. Kimoto-Nira et al. (2008) [33] reported changes to the immunomodulatory activity of a strain of *Bifidobacterium lactis* dependent on the type of fermentation broth used to grow the strain. Specifically, the interleukin-12, a cytokine which played a key role in the gut mucosal immune system response, was found to be enhanced in cell lines by *B*. *lactis* when grown on M17 fermentation media vs MRS media. In addition, the strains were found to have different cell wall compositions, at the sugar and fatty acid levels. Both the cell wall and immunomodulatory differences indicate that “sibling” strains of *B*. *lactis* grown using different technologies will yield bacteria with different clinical functionality.

Grześkowiak et al (2011) [5] have reported how the properties of probiotic *Lactobacillus rhamnosus* GG, may differ depending on the product and source of the strain, thus concluding that the original properties used in the selection of specific probiotic strains may be influenced by industrial production processes and, consequently, influence the outcome of human intervention studies. The examples above mentioned merely hint at the variables that can impact the functionality of a given probiotic. It is the reason why FDA requires for biosimilar “generic” biologics drugs comparative human clinical data in addition to *in vitro* comparative assays. In the case of the “original” VSL#3 formulation the complexity is further compounded by the fact that the product contains eight strains, which are all individually cultivated and lyophilized. Variations in their properties may affect the results of experimental studies as well clinical outcome of therapy interventions. In the present study, “original” and “newfound” VSL#3 formulations, both of them distributed under the “VSL#3” brand name in the UK, Holland and in Italy by the same company, were comparatively analyzed both for the bacterial composition and their ability to influence *in vitro* tumor cell lines. The results indicate significant differences between the two formulations in these properties under the same laboratory conditions. In our hands, the two formulations contained a significant different number of live and dead cells and differently influenced tumor cell viability, proliferation, cell cycle distribution and apoptosis level. Live viable probiotics seem to have superior efficacy to dead bacteria, both *in vivo* and *in vitro*, according to the literature [19] and gastrointestinal symptoms when dead bacteria are ingested have been reported [19].

Any changes brought to probiotic formulations and/or probiotic manufacturing processes should not be considered relevant and the product should be considered substantially equivalent, if they do not (a) compromise any original probiotic properties, (b) do not reduce the delivery of metabolically active, live bacteria, and, (c) do not change the effects of the product on epithelial and immune cell functions *in vitro*. According to our results obtained by comparing the “newfound” VSL#3 to the “original” VSL#3, this is not the case here.
