# Expression of Concern: Production Conditions Affect the *In Vitro* Anti-Tumoral Effects of a High Concentration Multi-Strain Probiotic Preparation

**DOI:** 10.1371/journal.pone.0212403

**Published:** 2019-02-07

**Authors:** The PLOS ONE Editors

The primary data supporting the results of this study [1] were not included with the article, and some results for lot 512058 were not reported. The authors have not agreed to share the dataset publicly, and so the article does not comply with PLOS’s Data Availability Policy.

Results reported in the article relied on experiments done using only one lot of “original” VSL#3 and one or two lots (depending on the experiment) of “newfound” VSL#3. Each experiment included only 2 replicates per condition. The authors indicated that the lots used were the only ones commercially available at the time the study was conducted.

Questions were raised post-publication about the statistical analysis methods reported in the article. The authors re-analysed the data using one-way or two-way ANOVA followed by post hoc Bonferroni tests, and a statistical reviewer advised that there was some redundancy in these analyses (e.g., treating live and dead cell percentages as separate factors in the ANOVA analysis) but that overall the results of the re-analyses supported the article’s conclusions. However, the authors have not agreed to publish the reanalysis files, and without a public dataset, the data analyses cannot be replicated independently.

The Competing Interests section of the article [1] is updated to include the following: Authors Cinque, La Torre, Lombardi, and Cifone were previously paid consultants for VSL Pharmaceuticals.

The authors contest this Competing Interests statement update and have noted that they disagree that the previous consultancies with VSL Pharmaceuticals fall within the scope of interests that must be declared per the journal’s editorial policy.

In light of the lack of compliance with the Data Availability policy, and to notify readers of the other issues noted above, the *PLOS ONE* Editors issue this Expression of Concern.
