# Correction: Production Conditions Affect the *In Vitro* Anti-Tumoral Effects of a High Concentration Multi-Strain Probiotic Preparation

**DOI:** 10.1371/journal.pone.0213134

**Published:** 2019-02-22

**Authors:** The PLOS ONE Editors

After the Expression of Concern was posted on this article [1, 2], the authors agreed to publish the underlying data and reanalysis files, which are provided in S1–S3 Files. Please view the files below.

This resolves the concern about data unavailability that motivated the editorial decision to post an Expression of Concern [2]. The correction made to the Competing Interests statement and the information about the study design discussed in [2] continue to apply.

## Supporting information

S1 File Data reanalysis report. Report of statistical analysis.
(PDF)

S2 File Prism files. PRISMA 5.0 reports.
(RAR)

S3 File Dataset. Global data.
(XLSX)
