# Supplementary material for: Correction: Production Conditions Affect the In Vitro Anti-Tumoral Effects of a High Concentration Multi-Strain Probiotic Preparation
Source: PLoS One. 2019 Feb 22;14(2):e0213134. doi: 10.1371/journal.pone.0213134 (PMC6386502; doi:10.1371/journal.pone.0213134)
Supplement: S1 File — Report of statistical analysis. (PDF) [file pone.0213134.s001.pdf]

ATTACHMENT #2 - REPORT OF STATISTICAL ANALYSIS

Below are described the statistical methods used for the analysis of data shown in the published article as well as the results of the new statistical analysis carried out with our statistical expert on data from **all performed experiments (ATTACHMENT #1)**.
All the reports Prism 5.0 are also attached in the folder named **ATTACHMENT #3**.

For each statistical analysis with t test are attached 3 files named TTEST#A-C followed by an identification code, as specified for each analysis below reported.
For each statistical analysis with ONE-WAY ANOVA + BONFERRONI POST HOC TEST is attached 1 file named OWA followed by an identification code, as specified for each analysis below reported.
For each statistical analysis with TWO-WAY ANOVA + BONFERRONI POST HOC TEST are attached 2 files named TWA#A-B followed by an identification code, as specified for each analysis below reported.

LIVE vs DEAD BACTERIA: The statistical analysis, primarily performed by using t test to compare mean values, has been now carried out with a two-way ANOVA test, followed by Bonferroni post hoc test with Prism 5.0 software. In particular, a two-way ANOVA with two factors: a) treatment (3 levels: TM091, 507132, 512058) and b) live/dead bacteria (2 levels: live, dead) was used to determine the significance of the difference in % live and dead bacteria between the groups. When P values of F-test were less than 0,05 (considered statistically significant) Bonferroni post-hoc analysis was performed.
The single reports generated by Prism 5.0 software are attached (**ATTACHMENT #3)** as specified in the table below.
Here in synthesis the results of both statistical analysis related to the experiment carried out (ten replicates) with the three probiotic sachets.

	t test	*TWO-WAY ANOVA followed by Bonferroni post hoc §*
ATTACHMENT #	***TTEST#A***	***TWA#A and TWA#B***
**507132 vs TM091**		
Live	*P<0,0001*	*P<0,001*
Dead	*P<0,0001*	*P<0,001*
ATTACHMENT #	***TTEST#B***	***TWA#A and TWA#B***
**507132 vs 512058**		
Live	*ns*	*ns*
Dead	*ns*	*ns*
ATTACHMENT #	***TTEST#C***	***TWA#A and TWA#B***
**TM091 vs 512058**		
Live	*P<0,0001*	*P<0,001*
Dead	*P<0,0001*	*P<0,001*

§OF NOTE*: *For PRISM 5.0 software, the upper limit for statistical significance with ANOVA test followed by Bonferroni post hoc is P<0,001

<u>TUMOR CELL VIABILITY:</u> The results shown in the published article were from the application of the repeated-measures (RM) two-way ANOVA test followed by Bonferroni post hoc test using Prism 5.0 software.
In particular, a two-way ANOVA-RM with two factors: a) treatment (7 levels: two formulations, TM091 and 507132, with 3 different concentrations, plus control) and b) time (3 levels: 12, 24 or 48 hrs), was used to determine the significance of the difference in cell number between the groups for the time trend. When the resulting differences were statistically significant (F-test with P<0,05), post-hoc analysis was performed using the Bonferroni test for pairwise comparisons between groups.
The single reports generated by Prism 5.0 software are attached (**<u>ATTACHMENT #3</u>**) as specified in the table below.
Here in synthesis the results of statistical analysis related to the three experiments carried out with the three used cell lines:

**Jurkat cells**	**22.2.2016**	**29.2.2016**	**7.3.2016**
ATTACHMENT #	***TWA#A Jurkat 1 Cell number and TWA#B Jurkat 1 Cell number***	***TWA#A Jurkat 2 Cell number and TWA#B Jurkat 2 Cell number***	***TWA#A Jurkat 3 Cell number and TWA#B Jurkat 3 Cell number***
**12 h**			
Control vs TM091 1 mg/ml	*ns*	*ns*	*ns*
Control vs TM091 5 mg/ml	*ns*	*ns*	*ns*
Control vs TM091 10 mg/ml	*ns*	*ns*	*ns*
Control vs 507132 1 mg/ml	*ns*	*ns*	*ns*
Control vs 507132 5 mg/ml	*ns*	*ns*	*ns*
Control vs 507132 10 mg/ml	*ns*	*ns*	*ns*
TM091 1 mg/ml vs 507132 1 mg/ml	*ns*	*ns*	*ns*
TM091 5 mg/ml vs 507132 5 mg/ml	*ns*	*ns*	*ns*
TM091 10 mg/ml vs 507132 10 mg/ml	*ns*	*ns*	*ns*
**24h**			
Control vs TM091 1 mg/ml	*P<0,001*	*P<0,01*	*P<0,001*
Control vs TM091 5 mg/ml	*P<0,001*	*P<0,001*	*P<0,001*
Control vs TM091 10 mg/ml	*P<0,001*	*P<0,001*	*P<0,001*
Control vs 507132 1 mg/ml	*ns*	*ns*	*ns*
Control vs 507132 5 mg/ml	*ns*	*ns*	*ns*
Control vs 507132 10 mg/ml	*P<0,001*	*P<0,01*	*P<0,01*
TM091 1 mg/ml vs 507132 1 mg/ml	*P<0,01*	*P<0,01*	*P<0,001*
TM091 5 mg/ml vs 507132 5 mg/ml	*P<0,01*	*P<0,01*	*P<0,001*
TM091 10 mg/ml vs 507132 10 mg/ml	*P<0,05*	*ns*	*P<0,001*
**48h**			
Control vs TM091 1 mg/ml	*P<0,001*	*P<0,001*	*P<0,001*
Control vs TM091 5 mg/ml	*P<0,001*	*P<0,001*	*P<0,001*
Control vs TM091 10 mg/ml	*P<0,001*	*P<0,001*	*P<0,001*
Control vs 507132 1 mg/ml	*ns*	*ns*	*P<0,001*
Control vs 507132 5 mg/ml	*P<0,001*	*P<0,001*	*P<0,001*
Control vs 507132 10 mg/ml	*P<0,001*	*P<0,001*	*P<0,001*
TM091 1 mg/ml vs 507132 1 mg/ml	*P<0,001*	*P<0,001*	*P<0,001*
TM091 5 mg/ml vs 507132 5 mg/ml	*P<0,001*	*P<0,001*	*P<0,001*
TM091 10 mg/ml vs 507132 10 mg/ml	*P<0,001*	*P<0,001*	*P<0,001*

**HT1080**	**15.2.2016**	**29.2.2016**	**7.3.2016**
ATTACHMENT #	***TWA#A HT1080 1 Cell number and TWA#B HT1080 1 Cell number***	***TWA#A HT1080 2 Cell number and TWA#B HT1080 2 Cell number***	***TWA#A HT1080 3 Cell number and TWA#B HT1080 3 Cell number***
**12h**			
Control vs TM091 1 mg/ml	*ns*	*ns*	*P<0,01*
Control vs TM091 5 mg/ml	*P<0,001*	*P<0,001*	*P<0,01*
Control vs TM091 10 mg/ml	*P<0,001*	*P<0,001*	*P<0,001*
Control vs 507132 1 mg/ml	*ns*	*ns*	*ns*
Control vs 507132 5 mg/ml	*P<0,001*	*ns*	*P<0,001*
Control vs 507132 10 mg/ml	*P<0,001*	*P<0,05*	*P<0,001*
TM091 1 mg/ml vs 507132 1 mg/ml	*P<0,01*	*ns*	*P<0,01*
TM091 5 mg/ml vs 507132 5 mg/ml	*ns*	*P<0,05*	*ns*
TM091 10 mg/ml vs 507132 10 mg/ml	*ns*	*ns*	*ns*
**24h**			
Control vs TM091 1 mg/ml	*P<0,05*	*ns*	*ns*
Control vs TM091 5 mg/ml	*P<0,001*	*P<0,01*	*P<0,001*
Control vs TM091 10 mg/ml	*P<0,001*	*P<0,001*	*P<0,001*
Control vs 507132 1 mg/ml	*ns*	*ns*	*ns*
Control vs 507132 5 mg/ml	*ns*	*ns*	*ns*
Control vs 507132 10 mg/ml	*P<0,01*	*ns*	*P<0,01*
TM091 1 mg/ml vs 507132 1 mg/ml	*ns*	*ns*	*ns*
TM091 5 mg/ml vs 507132 5 mg/ml	*P<0,01*	*P<0,05*	*P<0,01*
TM091 10 mg/ml vs 507132 10 mg/ml	*ns*	*ns*	*ns*
**48h**			
Control vs TM091 1 mg/ml	*P<0,001*	*P<0,001*	*P<0,001*
Control vs TM091 5 mg/ml	*P<0,001*	*P<0,001*	*P<0,001*
Control vs TM091 10 mg/ml	*P<0,001*	*P<0,001*	*P<0,001*
Control vs 507132 1 mg/ml	*P<0,001*	*P<0,001*	*P<0,001*
Control vs 507132 5 mg/ml	*P<0,001*	*P<0,001*	*P<0,001*
Control vs 507132 10 mg/ml	*P<0,001*	*P<0,001*	*P<0,001*
TM091 1 mg/ml vs 507132 1 mg/ml	*ns*	*P<0,05*	*ns*
TM091 5 mg/ml vs 507132 5 mg/ml	*P<0,001*	*P<0,01*	*P<0,001*
TM091 10 mg/ml vs 507132 10 mg/ml	*P<0,001*	*P<0,001*	*P<0,001*

**CACO-2**	**8.2.2016**	**22.3.2016**	**29.3.2016**
ATTACHMENT #	***TWA#A CACO-2 1 Cell number and TWA#B CACO-2 1 Cell number***	***TWA#A CACO-2 2 Cell number and TWA#B CACO-2 2 Cell number***	***TWA#A CACO-2 3 Cell number and TWA#B CACO-2 3 Cell number***
**12h**			
Control vs TM091 1 mg/ml	*ns*	*ns*	*Ns*
Control vs TM091 5 mg/ml	*P<0,001*	*P<0,05*	*P<0,001*
Control vs TM091 10 mg/ml	*P<0,001*	*P<0,001*	*P<0,001*
Control vs 507132 1 mg/ml	*ns*	*ns*	*Ns*
Control vs 507132 5 mg/ml	*ns*	*ns*	*Ns*
Control vs 507132 10 mg/ml	*P<0,05*	*ns*	*P<0,05*
TM091 1 mg/ml vs 507132 1 mg/ml	*ns*	*ns*	*Ns*
TM091 5 mg/ml vs 507132 5 mg/ml	*P<0,05*	*ns*	*P<0,001*
TM091 10 mg/ml vs 507132 10 mg/ml	*P<0,001*	*ns*	*P<0,001*
**24h**			
Control vs TM091 1 mg/ml	*P<0,001*	*P<0,001*	*P<0,001*
Control vs TM091 5 mg/ml	*P<0,001*	*P<0,001*	*P<0,001*
Control vs TM091 10 mg/ml	*P<0,001*	*P<0,001*	*P<0,001*
Control vs 507132 1 mg/ml	*P<0,05*	*ns*	*P<0,05*
Control vs 507132 5 mg/ml	*P<0,001*	*P<0,05*	*P<0,001*
Control vs 507132 10 mg/ml	*P<0,001*	*P<0,01*	*P<0,001*
TM091 1 mg/ml vs 507132 1 mg/ml	*ns*	*P<0,05*	*P<0,001*
TM091 5 mg/ml vs 507132 5 mg/ml	*ns*	*P<0,05*	*Ns*
TM091 10 mg/ml vs 507132 10 mg/ml	*P<0,05*	*P<0,001*	*P<0,05*
**48h**			
Control vs TM091 1 mg/ml	*P<0,001*	*P<0,01*	*P<0,001*
Control vs TM091 5 mg/ml	*P<0,001*	*P<0,01*	*P<0,001*
Control vs TM091 10 mg/ml	*P<0,001*	*P<0,001*	*P<0,001*
Control vs 507132 1 mg/ml	*ns*	*ns*	*P<0,05*
Control vs 507132 5 mg/ml	*ns*	*ns*	*Ns*
Control vs 507132 10 mg/ml	*P<0,01*	*ns*	*P<0,001*
TM091 1 mg/ml vs 507132 1 mg/ml	*P<0,001*	*P<0,05*	*P<0,001*
TM091 5 mg/ml vs 507132 5 mg/ml	*P<0,001*	*ns*	*P<0,001*
TM091 10 mg/ml vs 507132 10 mg/ml	*P<0,001*	*P<0,001*	*P<0,001*

<u>% DEAD CELLS:</u> The statistical analysis was primarily performed by using an t test using a PRISM 5.0. Statistics was re-analyzed again with the one-way ANOVA test followed by post hoc test Bonferroni using Prism 5.0 software, as suggested by our statistical expert. In particular, a one-way analysis of variance (ANOVA) with the one factor "treatment" (3 levels: Control, TM091, 507132. When P values of F-test were less than 0,05 (considered statistically significant) Bonferroni post-hoc analysis was performed.
The single reports generated by Prism 5.0 are attached (**<u>ATTACHMENT #3)</u>** as specified in the table below.
Here in synthesis the results of statistical analysis related to the three experiments carried out with the three used cell lines.

**JURKAT % Dead cells**						
	**22.2.2016**		**29.2.2016**		**7.3.2016**	
	**t test**	**One-way ANOVA + Bonferroni**	**t test**	**One-way ANOVA + Bonferroni**	**t test**	**One-way ANOVA + Bonferroni**
ATTACHMENT #	***TTEST#A-C Jurkat 1 % dead cells***	***OWA# Jurkat 1 % dead cells***	***TTEST#A-C Jurkat 2 % dead cells***	***OWA# Jurkat 2 % dead cells***	***TTEST#A-C Jurkat 3 % dead cells***	***OWA# Jurkat 3 % dead cells***
**Jurkat**						
Control vs TM091 10 mg/ml	*P<0,0001*	*P<0,001*	*P<0,001*	*P<0,001*	*P<0,005*	*P<0,01*
Control vs 507132 10 mg/ml	*P<0,0001*	*P<0,001*	*P<0,01*	*P<0,01*	*P<0,01*	*P<0,01*
TM091 10 mg/ml vs 507132 10 mg/ml	*P<0,0001*	*P<0,001*	*P<0,01*	*P<0,01*	*P<0,05*	*P<0,05*

**HT1080 % Dead cells**						
	**15.2.2016**		**29.2.2016**		**7.3.2016**	
	**t test**	**One-way ANOVA + Bonferroni**	**t test**	**One-way ANOVA + Bonferroni**	**t test**	**One-way ANOVA + Bonferroni**
ATTACHMENT #	***TTEST#A-C HT1080 1 % dead cells***	***OWA# HT1080 1 % dead cells***	***TTEST#A-C HT1080 2 % dead cells***	***OWA# HT1080 2 % dead cells***	***TTEST#A-C HT1080 3 % dead cells***	***OWA# HT1080 3 % dead cells***
**HT1080**						
Control vs TM091 10 mg/ml	*P<0,01*	*P<0,001*	*P<0,001*	*P<0,01*	*P<0,05*	*P<0,05*
Control vs 507132 10 mg/ml	*P<0,05*	*Ns*	*Ns*	*ns*	*ns*	*ns*
TM091 10 mg/ml vs 507132 10 mg/ml	*P<0,01*	*P<0,001*	*P<0,01*	*P<0,01*	*P<0,05*	*P<0,05*

**CACO-2 % Dead Cells**						
	**8.2.2016**		**21.3.2016**		**29.3.2016**	
	**t test**	**One-way ANOVA + Bonferroni**	**t test**	**One-way ANOVA + Bonferroni**	**t test**	**One-way ANOVA + Bonferroni**
ATTACHMENT #	***TTEST#A-C CACO-2 1 % dead cells***	***OWA# CACO-2 1 % dead cells***	***TTEST#A-C CACO-2 2 % dead cells***	***OWA# CACO-2 2 % dead cells***	***TTEST#A-C CACO-2 3 % dead cells***	***OWA# CACO-2 3 % dead cells***
**CACO-2**						
Control vs TM091 10 mg/ml	*P<0,05*	*P<0,01*	*P<0,005*	*P<0,001*	*P<0,01*	*P<0,01*
Control vs 507132 10 mg/ml	*P<0,05*	*Ns*	*P<0,01*	*P<0,01*	*P<0,05*	*P<0,05*
TM091 10 mg/ml vs 507132 10 mg/ml	*P<0,05*	*P<0,05*	*P<0,01*	*P<0,01*	*P<0,05*	*P<0,05*

COMPARISON BETWEEN TM091, 507132, 512058: Regarding the request of the Editor to show the data on the effects of the third sachet of probiotic, called 512058, not shown in the Fig. 3A and 3B of the article, **the results also as figures** are included in the file excel (**ATTACHMENT #1A**).
Also in this case, the two-way ANOVA-RM test followed by the Bonferroni post hoc test have been used to analyze data from **cell viability** experiments. In particular, a two-way ANOVA-RM with two factors: a) treatment (10 levels: 3 formulations, TM091, 507132, and 512058 with 3 different concentrations, plus control) and b) time (3 levels: 12, 24 or 48 hrs), was used to determine the significance of the difference in cell number between the groups for the time trend. When the resulting differences were statistically significant (F-test with P<0,05), post-hoc analysis was performed using the Bonferroni test for pairwise comparisons between groups.

Data from **% dead cells** were analyzed by Ordinary one-way ANOVA test followed by the Bonferroni post hoc test. In particular, one-way analysis of variance (ANOVA) with the one factor "treatment" (4 levels: Control, TM091, 507132, and 512058). When P values of F-test were less than 0,05 (considered statistically significant) Bonferroni post-hoc analysis was performed.
The single reports generated by Prism 5.0 software are attached (**ATTACHMENT #3)** as specified in the table below.
Here in synthesis the results of statistical analysis related to the experiment carried out with the three used cell lines:

**Cell viability**			
ATTACHMENT #	***TWA#A Jurkat 1+ Cell number and TWA#B Jurkat 1+ Cell number***	***TWA#A HT1080 1+ Cell number and TWA#B HT1080 1+ Cell number***	***TWA#A CACO-2 1+ Cell number and TWA#B CACO-2 1+ Cell number***
**12h**	**Jurkat**	**HT1080**	**CACO-2**
Control vs 512058 1 mg/ml	*ns*	*ns*	*ns*
Control vs 512058 5 mg/ml	*ns*	*P<0,001*	*P<0,05*
Control vs 512058 10 mg/ml	*ns*	*P<0,001*	*P<0,001*
Control vs TM091 1 mg/ml	*ns*	*ns*	*ns*
Control vs TM091 5 mg/ml	*ns*	*P<0,001*	*P<0,001*
Control vs TM091 10 mg/ml	*ns*	*P<0,001*	*P<0,001*
Control vs 507132 1 mg/ml	*ns*	*ns*	*ns*
Control vs 507132 5 mg/ml	*ns*	*P<0,001*	*ns*
Control vs 507132 10 mg/ml	*ns*	*P<0,001*	*P<0,05*
512058 1 mg/ml vs TM091 1 mg/ml	*ns*	*ns*	*ns*
512058 1 mg/ml vs 507132 1 mg/ml	*ns*	*ns*	*ns*
512058 5 mg/ml vs TM091 5 mg/ml	*ns*	*ns*	*ns*
512058 5 mg/ml vs 507132 5 mg/ml	*ns*	*ns*	*ns*
512058 10 mg/ml vs TM091 10 mg/ml	*ns*	*ns*	*P<0,05*
512058 10 mg/ml vs 507132 10 mg/ml	*ns*	*ns*	*P<0,05*
TM091 1 mg/ml vs 507132 1 mg/ml	*ns*	*P<0,01*	*ns*
TM091 5 mg/ml vs 507132 5 mg/ml	*ns*	*ns*	*P<0,01*
TM091 10 mg/ml vs 507132 10 mg/ml	*ns*	*ns*	*P<0,001*
**24h**	**Jurkat**	**HT1080**	**CACO-2**
Control vs 512058 1 mg/ml	*ns*	*ns*	*ns*
Control vs 512058 5 mg/ml	*ns*	*ns*	*P<0,001*
Control vs 512058 10 mg/ml	*P<0,001*	*P<0,05*	*P<0,001*
Control vs TM091 1 mg/ml	*P<0,001*	*P<0,05*	*P<0,001*

Control vs TM091 5 mg/ml	*P<0,001*	*P<0,001*	*P<0,001*
Control vs TM091 10 mg/ml	*P<0,001*	*P<0,001*	*P<0,001*
Control vs 507132 1 mg/ml	*ns*	*ns*	*P<0,01*
Control vs 507132 5 mg/ml	*ns*	*ns*	*P<0,001*
Control vs 507132 10 mg/ml	*P<0,001*	*P<0,01*	*P<0,001*
512058 1 mg/ml vs TM091 1 mg/ml	*P<0,001*	*ns*	*P<0,001*
512058 1 mg/ml vs 507132 1 mg/ml	*ns*	*ns*	*ns*
512058 5 mg/ml vs TM091 5 mg/ml	*P<0,001*	*P<0,001*	*ns*
512058 5 mg/ml vs 507132 5 mg/ml	*ns*	*ns*	*ns*
512058 10 mg/ml vs TM091 10 mg/ml	*P<0,01*	*ns*	*P<0,05*
512058 10 mg/ml vs 507132 10 mg/ml	*ns*	*ns*	*ns*
TM091 1 mg/ml vs 507132 1 mg/ml	*P<0,001*	*ns*	*P<0,05*
TM091 5 mg/ml vs 507132 5 mg/ml	*P<0,001*	*P<0,01*	*ns*
TM091 10 mg/ml vs 507132 10 mg/ml	*P<0,01*	*ns*	*P<0,01*
**48h**	**Jurkat**	**HT1080**	**CACO-2**
Control vs 512058 1 mg/ml	*P<0,001*	*P<0,001*	*ns*
Control vs 512058 5 mg/ml	*P<0,001*	*P<0,001*	*P<0,05*
Control vs 512058 10 mg/ml	*P<0,001*	*P<0,001*	*P<0,001*
Control vs TM091 1 mg/ml	*P<0,001*	*P<0,001*	*P<0,001*
Control vs TM091 5 mg/ml	*P<0,001*	*P<0,001*	*P<0,001*
Control vs TM091 10 mg/ml	*P<0,001*	*P<0,001*	*P<0,001*
Control vs 507132 1 mg/ml	*ns*	*P<0,001*	*ns*
Control vs 507132 5 mg/ml	*P<0,001*	*P<0,001*	*P<0,05*
Control vs 507132 10 mg/ml	*P<0,001*	*P<0,001*	*P<0,001*
512058 1 mg/ml vs TM091 1 mg/ml	*P<0,001*	*P<0,01*	*P<0,001*
512058 1 mg/ml vs 507132 1 mg/ml	*Ns*	*ns*	*ns*
512058 5 mg/ml vs TM091 5 mg/ml	*P<0,001*	*P<0,001*	*P<0,001*
512058 5 mg/ml vs 507132 5 mg/ml	*ns*	*ns*	*ns*
512058 10 mg/ml vs TM091 10 mg/ml	*P<0,001*	*P<0,001*	*P<0,001*
512058 10 mg/ml vs 507132 10 mg/ml	*P<0,001*	*P<0,001*	*ns*
TM091 1 mg/ml vs 507132 1 mg/ml	*P<0,001*	*ns*	*P<0,001*
TM091 5 mg/ml vs 507132 5 mg/ml	*P<0,001*	*P<0,001*	*P<0,001*
TM091 10 mg/ml vs 507132 10 mg/ml	*P<0,001*	*P<0,001*	*P<0,001*
**% Dead cells**			
	**Jurkat**	**HT1080**	**CACO-2**
	**One-way ANOVA + Bonferroni**	**One-way ANOVA + Bonferroni**	**One-way ANOVA + Bonferroni**
ATTACHMENT #	***OWA# Jurkat 1+ % Dead cells***	***OWA# HT1080 1+ % Dead cells***	***OWA# CACO-2 1+ % Dead cells***
Control vs 512058 10 mg/ml	*P<0,001*	*ns*	*ns*
Control vs TM091 10 mg/ml	*P<0,001*	*P<0,001*	*P<0,01*
Control vs 507132 10 mg/ml	*P<0,001*	*ns*	*ns*
512058 10 mg/ml vs TM091 10 mg/ml	*P<0,001*	*P<0,001*	*P<0,01*
512058 10 mg/ml vs 507132 10 mg/ml	*P<0,001*	*ns*	*ns*
TM091 10 mg/ml vs 507132 10 mg/ml	*P<0,001*	*P<0,001*	*P<0,01*

% WOUND CLOSURE: The results reported in the article were from the application of the Ordinary two-way ANOVA test followed by Bonferroni post hoc test Using a PRISM 5.0.

According to the suggestion of our statistical expert, data have also been re-analyzed using the Ordinary one-way ANOVA test followed by Bonferroni post hoc test with Prism 5.0. In particular, a one-way analysis of variance (ANOVA) with the one factor “treatment” (7 levels: 2 formulations (TM091, 507132) with 3 different concentrations, plus control). When P values of F-test were less than 0,05 (considered statistically significant) Bonferroni post-hoc analysis was performed.
The single reports from Prism 5.0 software are attached (**ATTACHMENT #3)** as specified in the table below.
Here in synthesis the results of both statistical analysis related to the three experiments carried out with the HT1080 and CACO-2 cells:

**HT1080 % Wound closure**						
	**14.3.2016**		**11.4.2016**		**26.4.2016**	
	**Two-way ANOVA + Bonferroni**	**One-way ANOVA + Bonferroni**	**Two-way ANOVA + Bonferroni**	**One-way ANOVA + Bonferroni**	**Two-way ANOVA + Bonferroni**	**One-way ANOVA + Bonferroni**
ATTACHMENT #	***TWA#A-B HT1080 1 %WH***	***OWA# HT1080 1 %WH***	***TWA#A-B HT1080 2 %WH***	***OWA# HT1080 2 %WH***	***TWA#A-B HT1080 3 %WH***	***OWA# HT1080 3 %WH***
**1 mg/ml**						
Control vs TM091	*P<0,001*	*P<0,001*	*P<0,001*	*P<0,01*	*P<0,001*	*P<0,001*
Control vs 507132	*Ns*	*ns*	*ns*	*ns*	*ns*	*ns*
TM091 vs 507132	*P<0,001*	*P<0,01*	*P<0,01*	*P<0,01*	*P<0,001*	*P<0,001*
**5 mg/ml**						
Control vs TM091	*P<0,001*	*P<0,001*	*P<0,001*	*P<0,001*	*P<0,001*	*P<0,001*
Control vs 507132	*Ns*	*ns*	*ns*	*ns*	*ns*	*ns*
TM091 vs 507132	*P<0,001*	*P<0,001*	*P<0,001*	*P<0,001*	*P<0,001*	*P<0,001*
**10 mg/ml**						
Control vs TM091	*P<0,001*	*P<0,001*	*P<0,001*	*P<0,001*	*P<0,001*	*P<0,001*
Control vs 507132	*ns*	*ns*	*ns*	*ns*	*P<0,05*	*ns*
TM091 vs 507132	*P<0,001*	*P<0,001*	*P<0,001*	*P<0,001*	*P<0,001*	*P<0,001*

CACO2 % Wound closure						
	**21.3.2016**		**29.3.2016**		**18.4.2016**	
	**Two-way ANOVA + Bonferroni**	**One-way ANOVA + Bonferroni**	**Two-way ANOVA + Bonferroni**	**One-way ANOVA + Bonferroni**	**Two-way ANOVA + Bonferroni**	**One-way ANOVA + Bonferroni**
ATTACHMENT #	***TWA#A-B CACO-2 1 %WH***	***OWA# CACO-2 1 %WH***	***TWA#A-B CACO-2 2 %WH***	***OWA# CACO-2 2 %WH***	***TWA#A-B CACO-2 3 %WH***	***OWA# CACO-2 3 %WH***
**1 mg/ml**						
Control vs TM091	*P<0,01*	*ns*	*P<0,001*	*P<0,05*	*P<0,01*	*ns*
Control vs 507132	*ns*	*ns*	*P<0,05*	*ns*	*ns*	*ns*
TM091 vs 507132	*P<0,05*	*ns*	*P<0,001*	*P<0,01*	*P<0,05*	*ns*
**5 mg/ml**						
Control vs TM091	*P<0,001*	*P<0,001*	*P<0,001*	*P<0,001*	*P<0,001*	*P<0,001*
Control vs 507132	*P<0,05*	*ns*	*ns*	*ns*	*P<0,05*	*ns*
TM091 vs 507132	*P<0,001*	*P<0,01*	*P<0,001*	*P<0,001*	*P<0,001*	*P<0,01*
**10 mg/ml**						
Control vs TM091	*P<0,001*	*P<0,001*	*P<0,001*	*P<0,01*	*P<0,001*	*P<0,001*
Control vs 507132	*ns*	*ns*	*P<0,05*	*ns*	*ns*	*ns*
TM091 vs 507132	*P<0,001*	*P<0,01*	*P<0,001*	*P<0,05*	*P<0,001*	*P<0,01*

CELL CYCLE ANALYSIS: The results reported in the article were from the application of the Ordinary two-way ANOVA test followed by Bonferroni post hoc test Using a PRISM 5.0.
In particular, a two-way ANOVA with two factors: a) treatment (3 levels: 2 formulations (TM091, 507132) plus control) and b) cell cycle phases (3 levels: G0/G1, S, G2/M), was used to determine the significance of the difference between the groups. When the resulting differences were statistically significant (F-test with $P<0,05$), post-hoc analysis was performed using the Bonferroni test for pairwise comparisons between groups. The single reports generated by Prism 5.0 software are attached (**ATTACHMENT #3)** as specified in the table below.
Here in synthesis the results of statistical analysis related to the three experiments carried out with the three used cell lines.

**JURKAT CELL CYCLE ANALYSES**			
	**5.4.2016**	**12.4.2016**	**19.4.2016**
	**TWO-WAY ANOVA + Bonferroni**	**TWO-WAY ANOVA + Bonferroni**	**TWO-WAY ANOVA + Bonferroni**
ATTACHMENT #	***TWA#A-B Jurkat 1 Cell cycle***	***TWA#A-B Jurkat 2 Cell cycle***	***TWA#A-B Jurkat 3 Cell cycle***
**Jurkat**			
**G0/G1 phase**			
Control vs TM091 10 mg/ml	*P<0,01*	*P<0,01*	*P<0,01*
Control vs 507132 10 mg/ml	*ns*	*ns*	*ns*
TM091 10 mg/ml vs 507132 10 mg/ml	*P<0,01*	*P<0,05*	*P<0,01*
**S phase**			
Control vs TM091 10 mg/ml	*ns*	*ns*	*ns*
Control vs 507132 10 mg/ml	*ns*	*ns*	*ns*
TM091 10 mg/ml vs 507132 10 mg/ml	*ns*	*ns*	*ns*
**G2/M phase**			
Control vs TM091 10 mg/ml	*P<0,001*	*P<0,001*	*P<0,01*
Control vs 507132 10 mg/ml	*ns*	*ns*	*ns*
TM091 10 mg/ml vs 507132 10 mg/ml	*P<0,01*	*P<0,01*	*P<0,05*

HT1080 CELL CYCLE ANALYSES			
	**15.3.2016**	**12.4.2016**	**27.4.2016**
	**TWO-WAY ANOVA + Bonferroni**	**TWO-WAY ANOVA + Bonferroni**	**TWO-WAY ANOVA + Bonferroni**
ATTACHMENT #	***TWA#A-B HT1080 1 Cell cycle***	***TWA#A-B HT1080 2 Cell cycle***	***TWA#A-B HT1080 3 Cell cycle***
**HT1080**			
**G0/G1**			
Control vs TM091 10 mg/ml	*P<0,001*	*P<0,001*	*P<0,01*
Control vs 507132 10 mg/ml	*P<0,001*	*P<0,001*	*P<0,001*
TM091 10 mg/ml vs 507132 10 mg/ml	*P<0,05*	*ns*	*ns*
**S**			
Control vs TM091 10 mg/ml	*P<0,001*	*P<0,001*	*P<0,001*
Control vs 507132 10 mg/ml	*P<0,001*	*P<0,001*	*P<0,001*
TM091 10 mg/ml vs 507132 10 mg/ml	*P<0,001*	*P<0,001*	*P<0,001*
**G2/M**			
Control vs TM091 10 mg/ml	*P<0,001*	*P<0,001*	*P<0,001*
Control vs 507132 10 mg/ml	*P<0,05*	*ns*	*ns*
TM091 10 mg/ml vs 507132 10 mg/ml	*P<0,001*	*P<0,001*	*P<0,001*

CACO-2 CELL CYCLE ANALYSES			
	**22.3.2016**	**30.3.2016**	**19.4.2016**
	**TWO-WAY ANOVA + Bonferroni**	**TWO-WAY ANOVA + Bonferroni**	**TWO-WAY ANOVA + Bonferroni**
ATTACHMENT #	***TWA#A-B CACO-2 1 Cell cycle***	***TWA#A-B CACO-2 2 Cell cycle***	***TWA#A-B CACO-2 3 Cell cycle***
**CACO-2**			
**G0/G1**			
Control vs TM091 10 mg/ml	*ns*	*ns*	*ns*
Control vs 507132 10 mg/ml	*ns*	*ns*	*ns*
TM091 10 mg/ml vs 507132 10 mg/ml	*ns*	*ns*	*ns*
**S**			
Control vs TM091 10 mg/ml	*P<0,001*	*P<0,001*	*P<0,01*
Control vs 507132 10 mg/ml	*ns*	*ns*	*ns*
TM091 10 mg/ml vs 507132 10 mg/ml	*P<0,01*	*P<0,01*	*P<0,01*
**G2/M**			
Control vs TM091 10 mg/ml	*P<0,01*	*P<0,05*	*P<0,01*
Control vs 507132 10 mg/ml	*ns*	*ns*	*ns*
TM091 10 mg/ml vs 507132 10 mg/ml	*P<0,05*	*P<0,05*	*P<0,05*

APOPTOSIS LEVEL (subdiploid peak): The results reported in the article were from the application of the t test using a PRISM 5.0 software.

As suggested by our expert, results have been re-analyzed using the Ordinary one-way ANOVA followed by Bonferroni post hoc test. In particular, a one-way analysis of variance (ANOVA) with the one factor "treatment" (3 levels: 2 formulations (TM091, 507132) plus control). When P values of F-test were less than 0,05 (considered statistically significant) Bonferroni post-hoc analysis was performed.

The single reports generated by Prism 5.0 software are attached (**ATTACHMENT #3**) as specified in the table below.

Here in synthesis the results of both statistical analysis related to the three experiments carried out with the three used cell lines.

% APOPTOTIC CELLS						
	**5.4.2016**		**12.4.2016**		**19.4.2016**	
	**t test**	**One-way ANOVA + Bonferroni**	**t test**	**One-way ANOVA + Bonferroni**	**t test**	**One-way ANOVA + Bonferroni**
ATTACHMENT #	***TTEST#A-C Jurkat 1 Apoptosis***	***OWA# Jurkat 1 Apoptosis***	***TTEST#A-C Jurkat 2 Apoptosis***	***OWA# Jurkat 2 Apoptosis***	***TTEST#A-C Jurkat 3 Apoptosis***	***OWA# Jurkat 3 Apoptosis***
**Jurkat**						
Control vs TM091 10 mg/ml	*P<0,01*	*P<0,01*	*P<0,01*	*P<0,01*	*P<0,01*	*P<0,01*
Control vs 507132 10 mg/ml	*P<0,05*	*P<0,05*	ns	ns	*P<0,01*	*P<0,05*
TM091 10 mg/ml vs 507132 10 mg/ml	*P<0,05*	*P<0,05*	*P<0,05*	*P<0,05*	*P<0,05*	*P<0,05*
	**15.3.2016**		**12.4.2016**		**27.4.2016**	
	**t test**	**One-way ANOVA + Bonferroni**	**t test**	**One-way ANOVA + Bonferroni**	**t test**	**One-way ANOVA + Bonferroni**
ATTACHMENT #	***TTEST#A-C HT1080 1 Apoptosis***	***OWA# HT1080 1 Apoptosis***	***TTEST#A-C HT1080 2 Apoptosis***	***OWA# HT1080 2 Apoptosis***	***TTEST#A-C HT1080 3 Apoptosis***	***OWA# HT1080 3 Apoptosis***
**HT1080**						
Control vs TM091 10 mg/ml	*P<0,01*	*P<0,01*	*P<0,01*	*P<0,01*	*P<0,01*	*P<0,01*
Control vs 507132 10 mg/ml	*P<0,01*	*P<0,01*	*P<0,05*	*P<0,05*	*P<0,05*	*P<0,05*
TM091 10 mg/ml vs 507132 10 mg/ml	*P<0,05*	*P<0,05*	*P<0,05*	*P<0,05*	*P<0,05*	*P<0,05*
	**22.3.2016**		**30.3.2016**		**19.4.2016**	
	**t test**	**One-way ANOVA + Bonferroni**	**t test**	**One-way ANOVA + Bonferroni**	**t test**	**One-way ANOVA + Bonferroni**
ATTACHMENT #	***TTEST#A-C CACO-2 1 Apoptosis***	***OWA# CACO-2 1 Apoptosis***	***TTEST#A-C CACO-2 2 Apoptosis***	***OWA# CACO-2 2 Apoptosis***	***TTEST#A-C CACO-2 3 Apoptosis***	***OWA# CACO-2 3 Apoptosis***
**CACO-2**						
Control vs TM091 10 mg/ml	*P<0,05*	*P<0,01*	*P<0,01*	*P<0,001*	*P<0,01*	*P<0,01*
Control vs 507132 10 mg/ml	*P<0,01*	*P<0,05*	*P<0,01*	*P<0,01*	*P<0,05*	*P<0,01*
TM091 10 mg/ml vs 507132 10 mg/ml	*P<0,05*	*P<0,05*	*P<0,01*	*P<0,01*	*P<0,05*	*P<0,05*

APOPTOSIS LEVEL (SYTO-13 STAINING): The results reported in the article were from the application of the t test using a PRISM 5.0.
As suggested by our expert, statistics was re-analyzed with Prism 5.0 using Ordinary one-way ANOVA followed by Bonferroni post hoc test. In particular, a one-way analysis of variance (ANOVA) with the one factor "treatment" (3 levels: 2 formulations (TM091, 507132) plus control). When P values of F-test were less than 0,05 (considered statistically significant) Bonferroni post-hoc analysis was performed.
The single reports generated by Prism 5.0 software are attached (**ATTACHMENT #3)** as specified in the table below.
Here in synthesis the results of both statistical analysis related to the experiment carried out with the three used cell lines.

**APOPTOSIS ANALYSIS WITH SYTO-13**		
	**16.5.2016**	
	**T test**	**ONE-WAY ANOVA + Bonferroni**
ATTACHMENT #	***TTEST#A-C Jurkat 1 Syto-13***	***OWA# Jurkat 1 Syto-13***
**Jurkat**		
Control vs TM091 10 mg/ml	*P<0,01*	*P<0,01*
Control vs 507132 10 mg/ml	*ns*	*ns*
TM091 10 mg/ml vs 507132 10 mg/ml	*P<0,01*	*P<0,01*
	**16.5.2016**	
	**T test**	**ONE-WAY ANOVA + Bonferroni**
ATTACHMENT #	***TTEST#A-C HT1080 1 Syto-13***	***OWA# HT1080 1 Syto-13***
**HT1080**		
Control vs TM091 10 mg/ml	*P<0,01*	*P<0,001*
Control vs 507132 10 mg/ml	*P<0,05*	*P<0,05*
TM091 10 mg/ml vs 507132 10 mg/ml	*P<0,01*	*P<0,01*
	**16.5.2016**	
	**T test**	**ONE-WAY ANOVA + Bonferroni**
ATTACHMENT #	***TTEST#A-C CACO-2 1 Syto-13***	***OWA# CACO-2 1 Syto-13***
**CACO-2**		
Control vs TM091 10 mg/ml	*P<0,01*	*P<0,001*
Control vs 507132 10 mg/ml	*P<0,01*	*ns*
TM091 10 mg/ml vs 507132 10 mg/ml	*P<0,01*	*P<0,001*

APOPTOSIS LEVEL (oligonucleosome assay): The results reported in the article were from the application of the t test using a PRISM 5.0.
As suggested by our expert, statistics was re-analyzed with Prism 5.0 using the Ordinary one-way ANOVA followed by Bonferroni post hoc test. In particular, a one-way analysis of variance (ANOVA) with the one factor "treatment" (3 levels: 2 formulations (TM091, 507132) plus control). When P values of F-test were less than 0,05 (considered statistically significant) Bonferroni post-hoc analysis was performed.
The single reports generated by Prism 5.0 software are attached (**ATTACHMENT #3**) as specified in the table below.
Here in synthesis the results of both statistical analysis related to the three experiments carried out with the three used cell lines.

OLIGONUCLEOSOME ASSAY						
	**2.5.2016**		**9.5.2016**		**16.5.2016**	
	**t test**	**One-way ANOVA + Bonferroni**	**t test**	**One-way ANOVA + Bonferroni**	**t test**	**One-way ANOVA + Bonferroni**
ATTACHMENT #	***TTEST#A-C Jurkat 1 Oligo***	***OWA# Jurkat 1 Oligo***	***TTEST#A-C Jurkat 2 Oligo***	***OWA# Jurkat 2 Oligo***	***TTEST#A-C Jurkat 3 Oligo***	***OWA# Jurkat 3 Oligo***
**Jurkat 48h**						
Control vs TM091 10 mg/ml	*P<0,01*	*P<0,01*	*P<0,01*	*P<0,01*	*P<0,01*	*P<0,01*
Control vs 507132 10 mg/ml	*ns*	*ns*	*ns*	*ns*	*ns*	*ns*
TM091 10 mg/ml vs 507132 10 mg/ml	*P<0,01*	*P<0,01*	*P<0,01*	*P<0,01*	*P<0,05*	*P<0,01*
	**2.5.2016**		**9.5.2016**		**16.5.2016**	
	**t test**	**One-way ANOVA + Bonferroni**	**t test**	**One-way ANOVA + Bonferroni**	**t test**	**One-way ANOVA + Bonferroni**
ATTACHMENT #	***TTEST#A-C HT1080 1 Oligo***	***OWA# HT1080 1 Oligo***	***TTEST#A-C HT1080 2 Oligo***	***OWA# HT1080 2 Oligo***	***TTEST#A-C HT1080 3 Oligo***	***OWA# HT1080 3 Oligo***
**HT1080 48h**						
Control vs TM091 10 mg/ml	*P<0,01*	*P<0,01*	*P<0,01*	*P<0,01*	*P<0,05*	*P<0,01*
Control vs 507132 10 mg/ml	ns	ns	ns	ns	*P<0,05*	ns
TM091 10 mg/ml vs 507132 10 mg/ml	*P<0,05*	*P<0,01*	*P<0,05*	*P<0,01*	*P<0,05*	*P<0,05*
	**2.5.2016**		**9.5.2016**		**16.5.2016**	
	**t test**	**One-way ANOVA + Bonferroni**	**t test**	**One-way ANOVA + Bonferroni**	**t test**	**One-way ANOVA + Bonferroni**
ATTACHMENT #	***TTEST#A-C CACO-2 1 Oligo***	***OWA# CACO-2 1 Oligo***	***TTEST#A-C CACO-2 2 Oligo***	***OWA# CACO-2 2 Oligo***	***TTEST#A-C CACO-2 3 Oligo***	***OWA# CACO-2 3 Oligo***
**CACO-2 48h**						
Control vs TM091 10 mg/ml	*P<0,05*	*P<0,05*	*P<0,05*	*P<0,01*	*P<0,01*	*P<0,001*
Control vs 507132 10 mg/ml	*ns*	*ns*	*P<0,05*	*ns*	*ns*	*P<0,05*
TM091 10 mg/ml vs 507132 10 mg/ml	*P<0,05*	*P<0,05*	*P<0,05*	*P<0,05*	*P<0,001*	*P<0,01*
